# Supplementary material for: Effects of Caprylic Acid and Eicosapentaenoic Acid on Lipids, Inflammatory Levels, and the JAK2/STAT3 Pathway in ABCA1-Deficient Mice and ABCA1 Knock-Down RAW264.7 Cells
Source: Nutrients. 2023 Mar 6;15(5):1296. doi: 10.3390/nu15051296 (PMC10005197; doi:10.3390/nu15051296)
Supplement: Supplementary file 1 [file nutrients-15-01296-s001.zip › Tables S1, S2, S3.pdf]

Table S1. Compositions of experimental diets (g/kg).

Ingredients	C8:0 diet	C16:0 diet	EPA diet	HFD diet
Corn starch	308.80	308.80	308.80	315.10
Casein	155.40	155.40	155.40	158.60
L-Cystine	2.33	2.33	2.33	2.38
Fiber(cellulose)	38.85	38.85	38.85	39.64
Sucrose	77.70	77.70	77.70	79.29
Maltose dextrin	102.60	102.60	102.60	104.69
AIN mineral mix 93G	27.20	27.20	27.20	27.76
AIN vitamin mix 93	7.77	7.77	7.77	7.93
Choline bitartrate	1.94	1.94	1.94	1.98
Soybean oil	54.39	54.39	54.39	55.50
Tert-butylhydroquinone	0.01	0.01	0.01	0.01
Lard	100.00	100.00	100.00	102.04
Yolk powder	100.00	100.00	100.00	102.04
Bile salt	3.00	3.00	3.00	3.06
C8:0	20.0	0.00	0.00	0.00
C16:0	0.00	20.0	0.00	0.00
EPA	0.00	0.00	20.00	0.00
Energy (kJ/g)	18.44	18.44	18.44	18.05
Protein	18.01	18.01	18.01	18.38
Fat	19.42	19.42	19.42	17.78
Carbohydrate	48.47	48.47	48.47	49.46
Cholesterol	0.029	0.029	0.029	0.030
Mineral mixture	0.078	0.078	0.078	0.080
Vitamin mixture	0.052	0.052	0.052	0.053
Fiber	0.150	0.150	0.150	0.153
Water	0.950	0.950	0.950	0.969
Others	0.106	0.106	0.106	0.108

C8:0, caprylic acid; C16:0, palmitic acid; EPA, eicosapentaenoic acid; HFD, high-fat diet.

Table S2. Fatty acid compositions of the experimental diets (g/kg).

Ingredients	C8:0 diet	C16:0 diet	EPA diet	HFD diet
C8:0	20.45	ND	ND	ND
C14:0	1.84	1.84	1.84	1.88
C16:0	43.36	63.36	43.36	44.24
C16:1	3.78	3.78	3.78	3.86
C18:0	21.88	21.88	21.88	22.33
C18:1	64.02	64.02	64.02	65.33
C18:2	34.87	34.87	34.87	35.58
C18:3	1.94	1.94	1.94	1.98
C20:0	0.41	0.41	0.41	0.42
C20:5	0.82	0.82	20.82	0.84
C22:0	0.31	0.31	0.31	0.32

C22:6	0.51	0.51	0.51	0.52

C8:0, caprylic acid; C14:0, myristic acid; C16:0, palmitic acid; C16:1, palmitoleic acid; C18:0, stearic acid; C18:1, oleic acid; C18:2, linoleic acid; C18:3, linolenic acid; C20:0, arachidic acid; C20:5, eicosapentaenoic acid, EPA; C22:0, behenic acid; C22:6, docosahexenoic acid, DHA; HFD, high-fat diet; ND, not detectable.

Table S3. qRT-PCR Primer sequences in plasmid siRNA of ABCA1.

Gene	Sense primer (5'-3')	Antisense primer (5'-3')
NC	UUCUCCGAACGUGUCACGUTT	ACGUGACACGUUCGGAGAATT
ABCA1-1530	GGAGUUGGCUGUGUUCCAUTT	AUGGAACACAGCCAACUCCTT
ABCA1-1701	CCAAGACAUCAUGGCGUUUTT	AAACGCCAUGAUGUCUUGGTT
ABCA1-4931	GGGUGUCAGUAAUUCUCAATT	UUGAGAAUUACUGACACCCTT

NC, normal control; ABCA1, ATP binding box transporter A1.
